# Tripartite hydrogen-bonding as a driving force for high-concentration cyclization of poly(l-lactide)

**DOI:** 10.1039/d5sc08070c

**Published:** 2025-12-23

**Authors:** Sébastien Moins, Alexandre Mignot, Céline Henoumont, Sophie Laurent, Philippe Leclère, Olivier Coulembier

**Affiliations:** a Laboratory of Polymeric and Composite Materials, University of Mons Place du Parc 23 7000 Mons Belgium olivier.coulembier@umons.ac.be; b Laboratory for Physics of Nanomaterials and Energy (LPNE), Research Institute for Materials Science and Engineering, CIRMAP, University of Mons Belgium; c General, Organic and Biomedical Chemistry, NMR and Molecular Imaging Laboratory, University of Mons Place du Parc 20 7000 Mons Belgium

## Abstract

A three-step strategy enables efficient cyclization of high-*M*_n_ PLLA at 0.5 M. Key to success is a persistent tripartite complex driven by hydrogen bonding and ion pairing. Selective intramolecular transesterification at a benzylic ester triggers ring closure, affording cyclic PLLA (*M*_n_ ≈ 26 000 g mol^−1^) under synthetically practical conditions. SPM imaging reveals nanorings with diameters consistent with the expected contour length of cyclic PLLA chains.

## Abstract 2

A three-step strategy enables efficient cyclization of high-*M*_n_ PLLA at 0.5 M.

## Graphical abstract

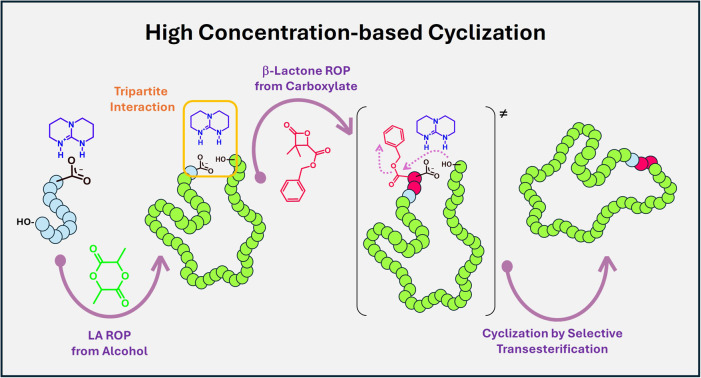

Cyclic polymers represent a compelling class of macromolecules with unique physical and chemical properties that arise from their closed-loop topology.^1–5^ The absence of chain ends reduces their hydrodynamic volume and alters chain entanglement and reptation dynamics, leading to distinctive behavior in solution, in the melt, and in confined systems. These characteristics hold promise for advanced applications in nanomedicine, electronics, and responsive materials.

Synthetic access to cyclic polymers typically relies on two strategies, *i.e.* ring-expansion polymerization (REP), in which a growing cyclic intermediate is propagated through successive monomer insertions, and ring-closure (RC), which involves the end-to-end cyclization of linear precursors.^6–8^ REP offers high molecular weights and often narrow dispersities, but requires specialized catalysts and is limited in scope. RC strategies are more general and compatible with diverse polymer chemistries, particularly when well-defined telechelics are used. However, RC is strongly entropically disfavored and traditionally requires ultra-dilute conditions (<10^−5^ M) to suppress intermolecular coupling and ensure intramolecular cyclization, making the process inefficient, hard to scale, and difficult to apply to polymers beyond ∼25 kDa.^9^

To circumvent these challenges, Tezuka and co-workers developed an elegant bimolecular electrostatic self-assembly and covalent fixation strategy.^10^ By decorating the termini of poly(tetrahydrofuran) chains with strained *N*-phenylpyrrolidinium cations and reacting them with dianionic coupling agents, they achieved high-purity cyclic polymers in dilute solution. In this approach, long-range electrostatic interactions guide the two reactive chain ends into proximity through salt-pair formation, significantly accelerating the initial coupling event. Upon heating, the ionic pairs undergo covalent fixation, leading to permanent cyclic structures. This methodology not only enhances cyclization efficiency but also opens access to more complex architectures, including axially chiral, spirocyclic, and graph-like multicyclic topologies, by capitalizing on topological control during assembly.^11–14^

Building on this principle of electrostatically driven preorganization, more recent approaches have turned toward intramolecular folding of charged polymer chains into compact conformations prior to cyclization.^15^ A particularly notable example employed poly(2-(dimethylamino)ethyl methacrylate)-based telechelics that, in the presence of acid-based crosslinkers, form charged single-chain nanoparticles (SCNPs) in concentrated solution. The proximity of chain ends within these dynamic globules enabled efficient UV-triggered click cyclization at concentrations up to 150 mg mL^−1^ (∼0.5 M). While this represents a major step toward high-concentration ring-closure, the system remains highly specialized, relying on tailored architectures, delicate equilibria, and multi-step chemistry.

In this context, we report a conceptually distinct ring-closure strategy that also leverages electrostatic interactions, but in a simpler and more generalizable framework. Our approach enables cyclization at similarly high concentrations without relying on dynamic SCNP formation. The strategy emerges from a previous observation made in 2019, during investigations on simultaneous ring-opening polymerization (ROP) of four- and six-membered lactones. In that study, block and star-shaped copolymers were obtained in one-step from heterodifunctional initiators bearing hydroxyl and carboxylate functionalities.^16^ Notably, the carboxylate group selectively initiated the polymerization of four-membered β-lactones, while the hydroxyl group, activated through interaction with the carboxylate, was responsible for initiating the polymerization of six-membered lactones such as lactide (LA). Among the catalytic systems explored, the combination of 12-hydroxydodecanoic acid (HDA) and the organic base 1,5,7-triazabicyclo[4.4.0]dec-5-ene (TBD) proved particularly intriguing (Fig. 1a). While it effectively enabled the ROP of dimethylated benzyl β-malolactonate (dMMLABn) from the carboxylate end-group in DCM (Fig. 1b), it consistently led to products with broad dispersities and multimodal SEC traces when the reaction was allowed to proceed over extended times at a [dMMLABn]_0_ of 1.33 M. Detailed ^1^H NMR and MALDI-ToF analyses revealed that this lack of control originated mainly from intramolecular transesterification between the terminal hydroxyl group of the HDA and a pendant benzyl ester moieties, consistent with a backbiting-type ring-closure (Fig. 1c).

**Fig. 1:**
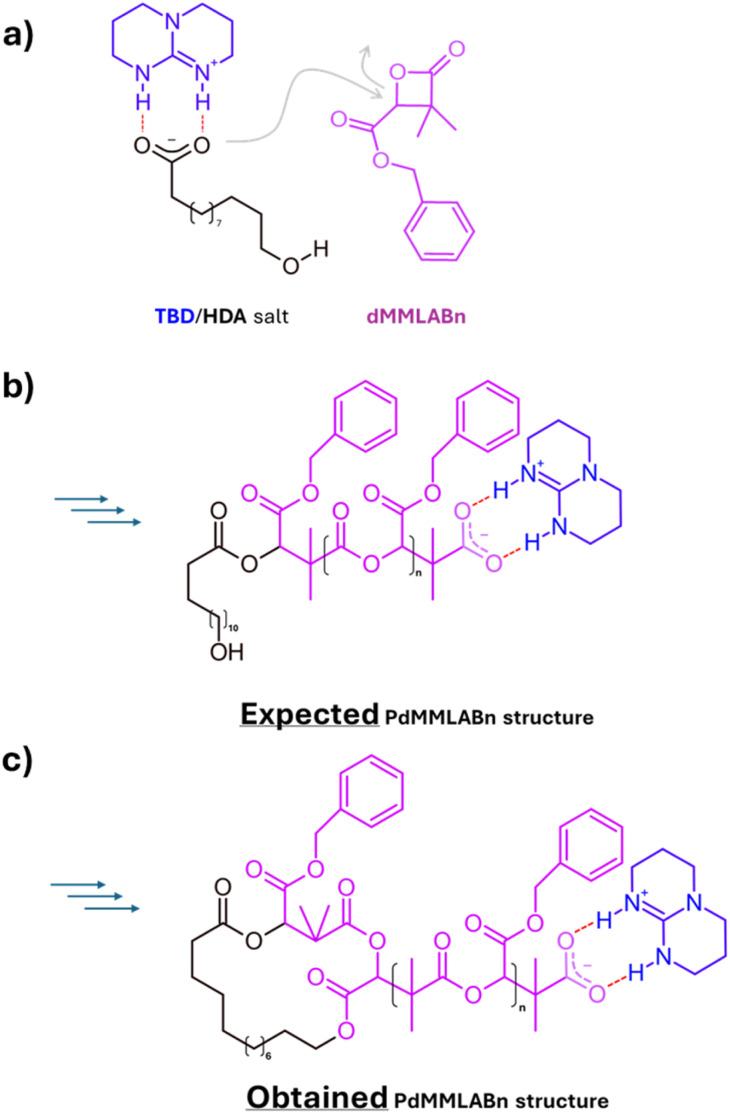
Previous work showing the origin of the ring-closure process. (a) Nucleophilic activation of dMMLABn by the HDA/TBD carboxylate salt. (b) Expected linear PdMMLABn chain. (c) Actual cyclized product formed through intramolecular transesterification (backbiting-type) between the terminal hydroxyl of HDA and a pendant benzyl ester group.

In the present study and to rationalize the intramolecular formation of cyclic structures observed in our previous work, we hypothesize that the terminal alcohol of the HDA should remain in close and continuous proximity to the catalytic center formed by the TBD.H^+^/carboxylate ion-pair end-group (Fig. 2 top left and S2). With a sufficiently strong interaction, even thorough a polymerization course, we might postulate a very limited exchange with the catalytic center of another polymer chain, strongly limiting intermolecular reactions.

**Fig. 2:**
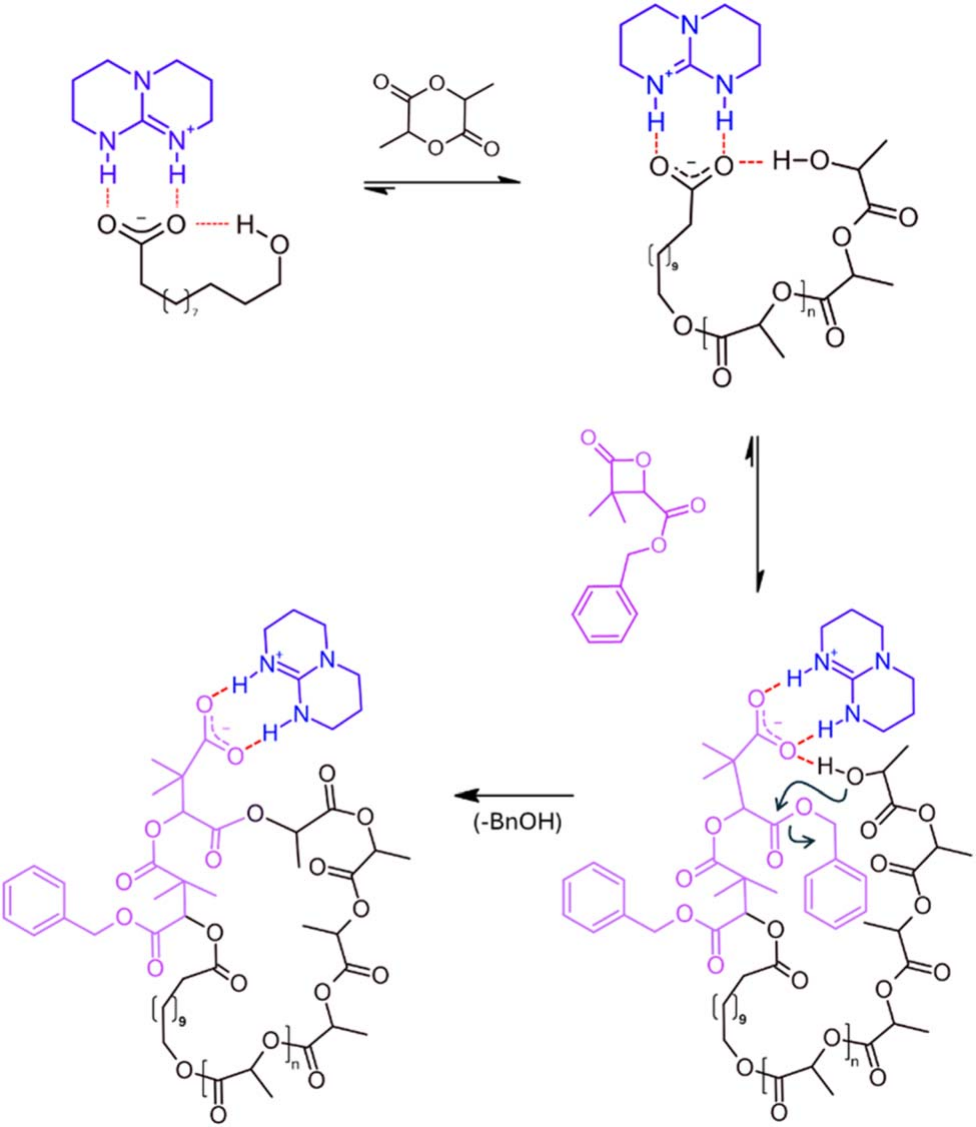
Three-step mechanism proposed for the formation of *cyclo*-PLA *via* intramolecular transesterification: (1) ROP of lactide initiated by a TBD/HDA salt, yielding a linear PLA with a terminal hydroxyl group in proximity to the catalytic centre; (2) insertion of 2 units of dMMLABn at the PLA carboxylate terminus; and (3) intramolecular transesterification, promoted by the spatial proximity of the hydroxyl group within the TBD.H^+^/carboxylate ion pair, resulting in ring closure.

Consistent with this hypothesis, ^1^H NMR spectroscopic evidence confirms the existence of stable hydrogen-bonded assemblies between the HDA and TBD in CDCl_3_ (Fig. S1). In presence of one equivalent of TBD, HDA methylene carboxy protons were most affected, as indicated by a change in chemical shift of the –CH_2_–CO_2_H resonance (Δ*δ* = 0.13 ppm). The formation of the TBD.H^+^-based carboxylate causes a downfield shift of the methylene hydroxy protons (Δ*δ* = 0.03 ppm), confirming the formation of a ternary assembly involving the TBD.H^+^/carboxylate ion pair and the hydroxyl group of the HDA. Crystallographic studies by Görbitz and Yadav further support this interpretation, providing compelling evidence that guanidine-based organocatalysts, such as TBD, form tightly bound complexes with carboxylic acids that can further associate with neutral hydrogen-bond donors.^17,18^ In particular, solid-state structures of 2 : 1 complexes between TBD and various carboxylic acids in the presence of a single water molecule revealed a well-defined tridentate hydrogen-bonded motif, in which the water bridges the two carboxylate oxygen atoms through strong, nearly linear O–H⋯O interactions. The observed hydrogen-bond distances (1.72–1.90 Å) and bonding angles (170–178°) correspond to robust association energies (−4 to −10 kcal mol^−1^), consistent with the formation of similarly stable tripartite assemblies in which the hydroxyl group of HDA could play a role analogous to that of the bridging water molecule. Although these structures involve water as the hydrogen-bond donor, a comparable geometry is expected to arise in solution between the TBD.H^+^/carboxylate pair and the hydroxyl end-group of the HDA (Fig. S2). Alcohols display similar hydrogen-bond donating abilities,^19^ and under the mildly solvating conditions employed here, the formation of a pseudo-cyclic [TBD.H^+^/RCOO^−^/ROH] complex appears not only feasible but also likely to persist over time. This spatial confinement would maintain the growing chain end preorganized near the catalytic centre, thereby facilitating selective intramolecular reactivity.

While intramolecular transfer reactions typically hinder the controlled synthesis of PdMMLABn, they may conversely offer an opportunity for the preparation of cyclic architectures, particularly in the context of cyclic poly(lactide) (*cyclo*-PLA), a topic our group has been exploring for some time.^20–29^ Our approach relies on a general mechanism proceeding through a three-step process (Fig. 2), in which the last two steps occur with distinct kinetics. At first, the polymerization of l-lactide (LLA) is initiated from a TBD/HDA salt, leading to the formation of a linear PLLA chain bearing a terminal hydroxyl group in close proximity to the catalytic salt.^16^ Upon completion of the LLA polymerization, the second step involves the addition of few units (2 to 3) of dMMLABn, allowing this lactone to insert at the carboxylate PLLA chain end. With the terminal alcohol still associated with the TBD.H^+^/carboxylate ion pair, a highly favorable geometry is established for intramolecular transesterification, which constitutes the third step. Notably, this final transformation proceeds more slowly than the preceding β-lactone insertion, triggering an efficient ring-closure.

The first step consisted then in evaluating the ability of the HDA/TBD system to initiate and control the ROP of LLA under conditions relevant to the proposed strategy, *i.e.*, in terms of monomer concentration and/or HDA-to-TBD ratio. In our previous work,^16^ using of an equimolar HDA-to-TBD led to a complete loss of control over the polymerization of a 2 M LLA solution in DCM (*Ɖ*_M_ = *M*_w_/*M*_n_ = 2 to 2.5). To tentatively improve control, this step was re-examined under slightly more dilute conditions (0.5 M in DCM), targeting a degree of polymerization (DP = [LLA]_0_/[HDA]_0_) of 40, and varying the [HDA]_0_/[TBD]_0_ ratio. At ratios of 10 and 5, no significant polymerization was observed even after 24 hours. By contrast, a [HDA]_0_/[TBD]_0_ ratio of 1 enabled a well-controlled polymerization, reaching 76% conversion within 30 minutes and yielding a PLLA with a relative number-average molar mass determined by size exclusion chromatography (*M*_n_, SEC) of 6900 g mol^−1^ and a *Ɖ*_M_ of 1.23.

The second step of the process was then evaluated by adding 2 equivalents of dMMLABn to the reaction mixture. This limited amount was chosen to introduce a few benzyl ester functionalities required for the third step, while minimizing the risk of forming well-defined diblock copolymers. To approach near-quantitative conversion of LLA prior to β-lactone insertion, the dMMLABn was added 60 minutes after initiation (conversion in LLA ∼94%). At 0.5 M in DCM, the insertion of the β-lactone from the carboxylate terminus was found to be exceedingly slow, requiring up to 164 hours for the incorporation of a single unit. Moreover, no evidence of transesterification at the benzyl ester function was observed by ^1^H NMR spectroscopy under these conditions. Since the overall objective consists on accessing cyclic structures under relatively concentrated conditions, the [LLA]_0_ was maintained at 0.5 M, but the [HDA]_0_/[TBD]_0_ ratio was slightly decreased to 0.91 to work with a slight excess of TBD regarding the HDA ([TBD]_0_ = 1.1 [HDA]_0_). Under these conditions, polymerization of LLA (DP = 40) reached full conversion within 15 minutes, affording a PLLA with *M*_n_, SEC = 7300 g mol^−1^, but with a truncated molar mass distribution evidenced by a slight shoulder, indicating that exceeding an TBD/HDA ratio of 1.1 may compromise control. Under those conditions, subsequent addition of 2 equivalents of dMMLABn to the as-obtained PLLA chains led to a notable decrease in the SEC-determined molecular weight, with *M*_n_, SEC dropping to 5200 g mol^−1^ (Fig. 3).

**Fig. 3:**
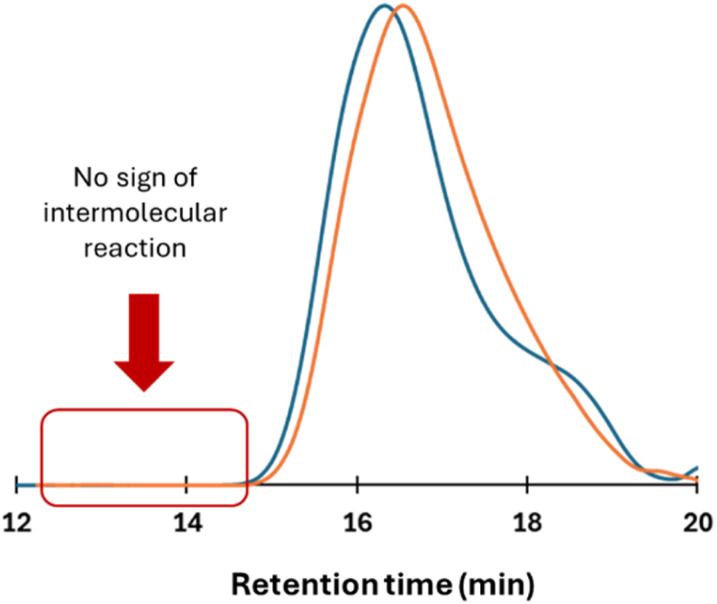
SEC traces of a linear PLLA sample obtained after 15 minutes of LLA polymerization from a HDA/TBD salt in DCM ([HDA]_0_/[TBD]_0_ = 1.1, [LLA]_0_ = 0.5 M, blue curve) and its cyclic homologue obtained after addition of 2 unities of dMMLABn in the same experimental conditions (orange curve).

Such a reduction in apparent molecular weight is consistent with the change in hydrodynamic volume typically observed when comparing linear and cyclic polymer architectures, assuming that cyclization has indeed occurred. Notably, no sign of chain reshuffling, *i.e.*, intermolecular transesterification, was detected at low retention times.

The same protocol was applied to the preparation of a higher molar mass PLLA, targeting a DP of 200. After 105 min of polymerization, SEC analysis revealed the formation of a PLLA with a relative *M*_n_, SEC of 45 000 g mol^−1^ (*Ɖ*_M_ = 1.16). Subsequent addition of circa three equivalents of dMMLABn, followed by 60 minutes of stirring, led to a marked decrease in the relative molar mass to 26 000 g mol^−1^, with no evidence of intermolecular coupling. Following precipitation into heptane, two distinct ^1^H NMR analyses were performed, *i.e.*, one on the dried polymer sample, and one on the supernatant solvent gently evaporated at 21 °C, over four days. As shown in Fig. 4, the ^1^H NMR spectrum of the evaporated precipitation medium displays a singlet at *δ* = 4.67 ppm, which unambiguously corresponds to the methylene protons of benzyl alcohol. This observation confirms the occurrence of a transesterification reaction between the isopropyl chain end of PLLA and one of the benzylic ester groups of the terminal dMMLABn unit incorporated at the PLLA extremity. Complementarily, in the polymer spectrum, this transformation is further evidenced by the disappearance of one benzylic signal among the three dMMLABn units (see SI for calculation from NMR integrations and Fig. S3), and the appearance of a new methine resonance at *δ* = 4.12 ppm, indicative of the newly formed ester linkage, as already observed in our previous study.^16^ For visual comparison, this signal is presented alongside the two methylene protons of the HDA moiety, which resonate at *δ* = 4.36 ppm, in a zoomed-in region between *δ* = 4.1 and 4.4 ppm.

**Fig. 4:**
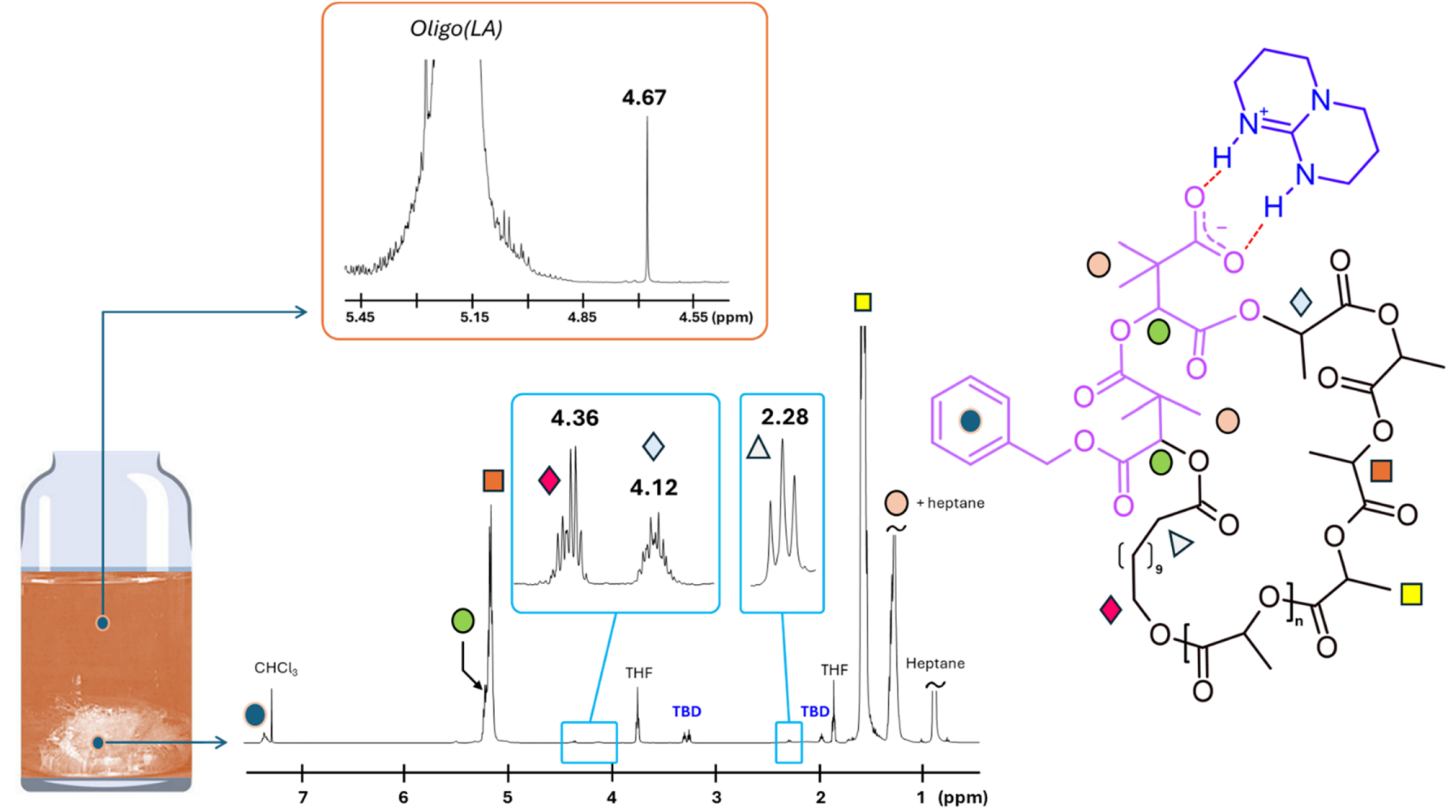
Evidence for chain-end transesterification and cyclization of PLLA (DP = 181) at 0.5 M. The ^1^H NMR spectrum of the evaporated precipitation solvent reveals a singlet at 4.67 ppm (benzyl alcohol). The spectrum of the isolated polymer shows a new methine signal at 4.12 ppm, consistent with ester formation, and HDA methylene protons at 4.36 ppm (zoomed region). A schematic vial image illustrates the precipitation process.

Interestingly, the preferential attack on the benzylic ester is not only driven by spatial proximity within the preorganized complex, but also by the inherently greater electrophilicity of the benzylic ester group. Early kinetic studies on ester hydrolysis reactions have shown that benzyl acetate undergoes base-catalyzed saponification with a second-order rate constant of 14 M^−1^ min^−1^, compared to only 1.84 M^−1^ min^−1^ for isopropyl acetate.^30^ This ∼7-fold rate enhancement underscores the intrinsic reactivity of benzyl esters and provides a further rationale for their selective involvement in intramolecular ring-closing transesterification.

Based on the integration of these signals and the number of lactidyl repeat units, an absolute molar mass of 25 850 g mol^−1^ was determined for the cyclic product. Notably, applying the Mark–Houwink parameters for linear PLLA prior to cyclization yielded a matching value of 30 600 g mol^−1^.^31^ This relatively good agreement supports both the occurrence of intramolecular ring closure and the reliability of the analytical approach. To the best of our knowledge, this constitutes the first reported example of successful cyclization for a polymer sample with *M*_n_ of 26 000 g mol^−1^ at a cyclization concentration of 0.5 M.

To verify that this structural rearrangement translates into the expected topological signature, DOSY NMR experiments were performed. As expected, the cyclic sample exhibited a noticeably higher diffusion coefficient than the linear analogue, reflecting its more compact hydrodynamic volume. The linear PLLA displayed a diffusion coefficient of *D* = 1.13 × 10^−11^ m^2^ s^−1^, whereas the cyclic sample diffused faster, with *D* = 1.09 × 10^−10^ m^2^ s^−1^ (Fig. S4). This increase in diffusion is fully consistent with the expected linear-to-cyclic contraction and aligns with previous DOSY studies used to discriminate polymer topologies of similar size.^32^

Cyclic PLLA rings were finally visualized by Scanning Probe Microscopy (SPM) in PeakForce Tapping mode (Fig. 5 and S5). A few microliters of a highly diluted chloroform solution (1.1 µg mL^−1^) were drop-cast onto freshly cleaned silicon wafers. At this concentration, isolated rings were clearly distinguishable. The topographic image (Fig. 5a–c) shows round features with an average diameter of ∼30 nm and an apparent height of ∼2 nm (Fig. 5d). In the corresponding adhesion map (Fig. S5b), the substrate (light blue) and the rings (dark blue) are well differentiated. Automated circle-detection analysis (Fig. S5c) identified nearly 200 objects, yielding an average diameter of 26 ± 1 nm. These dimensions are consistent with the theoretical size expected for the cyclic PLLA chains estimated above. Using the polymerization degree (DP) determined by NMR (*M*_n_ = 26 000 g mol^−1^; DP = 181) and a repeat projection of 0.5–0.6 nm per lactidyl unit, the geometric diameter expected for a fully extended circular conformation (*D* = *L*_c_/π) lies between 28.8 and 34.6 nm. The measured diameter therefore falls within the range expected for a cyclic chain of this molar mass once surface flattening, tip convolution and conformational variability upon adsorption are considered. The height profile (2 nm) is consistent with partial flattening/stacking of chain segments on the substrate rather than particulate contamination.

**Fig. 5:**
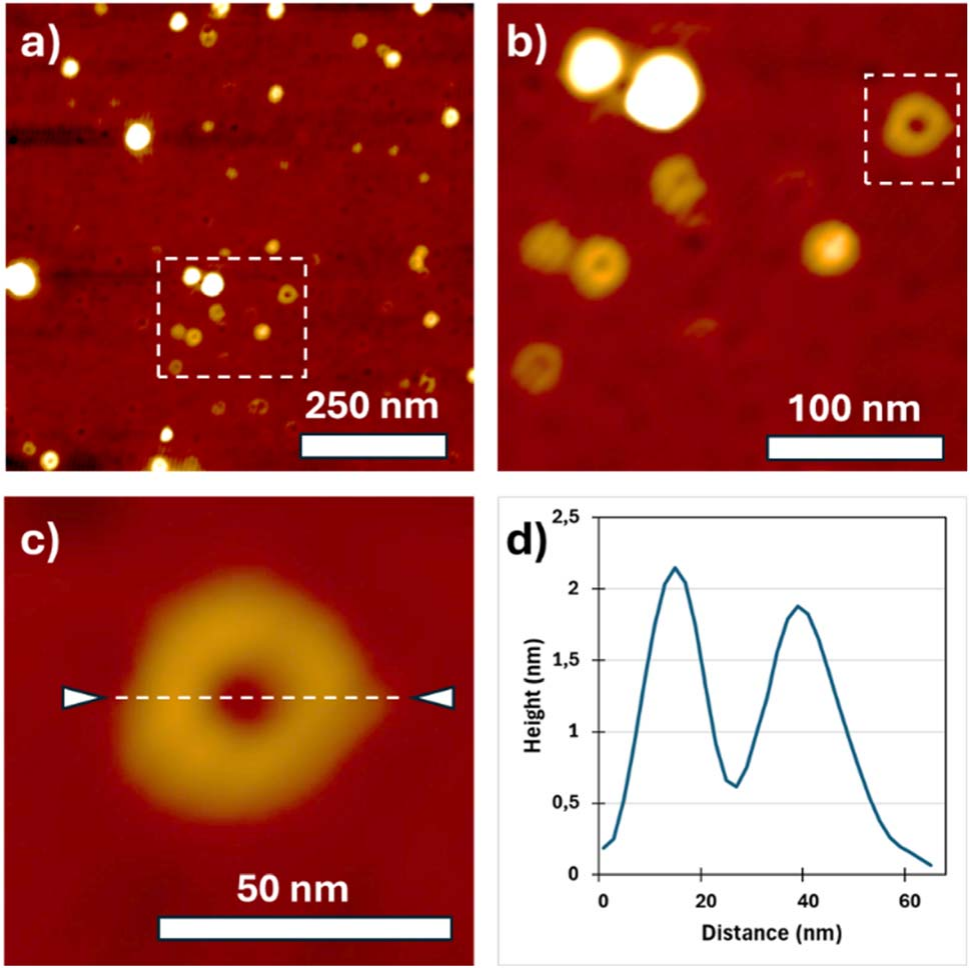
Scanning Probe Microscopy (SPM) acquisitions of a PLLA deposit from chloroform solution: (a–c) topography zooms, (d) height profile of Fig. 5c.

## Conclusions

This work establishes a mechanistic framework for achieving intramolecular cyclization of high-molecular-weight poly(l-lactide) in concentrated solution. By exploiting the persistent association between the TBD.H^+^/carboxylate ion pair and a terminal hydroxyl function, both polymer ends remain preorganized during polymerization, enabling a selective transesterification at an inserted benzylic ester to trigger ring closure. The reaction proceeds efficiently at 0.5 M and yields cyclic PLLA with *M*_n_ = 26 000 g mol^−1^, as confirmed by SEC, NMR and SPM imaging. Beyond providing the first demonstration of high-*M*_n_ PLLA cyclization under such conditions, this function-directed concept introduces a simple and generalizable design principle for cyclic polymer synthesis by maintaining end-group proximity through ion-pair preorganization.

## Author contributions

Olivier Coulembier conceptualized the project, performed the polymerization experiments and characterization (NMR, SEC) with Sébastien Moins, and wrote the manuscript. Alexandre Mignot and Philippe Leclère conducted the SPM acquisitions and contributed to data analysis interpretation. Céline Henoumont and Sophie Laurent performed the DOSY NMR experiments and contributed to data analysis interpretation. All authors discussed the results and contributed to the final version of the manuscript.

## Conflicts of interest

There are no conflicts to declare.

## Supplementary Material

SC-OLF-D5SC08070C-s001

## Data Availability

The data supporting this article have been included as part of the supplementary information (SI). Supplementary information is available. See DOI: https://doi.org/10.1039/d5sc08070c.
