# Supplementary material for: Tripartite hydrogen-bonding as a driving force for high-concentration cyclization of poly(l-lactide)
Source: Chem Sci. 2025 Dec 23;17(7):3804–9. doi: 10.1039/d5sc08070c (PMC12766472; doi:10.1039/d5sc08070c)
Supplement: SC-017-D5SC08070C-s001 [file SC-017-D5SC08070C-s001.pdf]

Supplementary Information (SI) for Chemical Science.
This journal is © The Royal Society of Chemistry 2025

SUPPORTING INFORMATION

…

Tripartite Hydrogen-Bonding as a Driving Force for High-Concentration Cyclization of Poly(L-lactide)

Sébastien Moins,[a] Alexandre Mignot,[b] Céline Henoumont,[c] Sophie Laurent,[c] Philippe Leclère,[b] Olivier Coulembier[a]*

a) Laboratory of Polymeric and Composite Materials (LPCM), Center of Innovation and Research in Materials and Polymers (CIRMAP), University of Mons, Place du Parc 20, 7000 Mons, Belgium.
b) Laboratory for Physics of Nanomaterials and Energy (LPNE) Research, Institute for Materials Science and Engineering, CIRMAP, University of Mons, Belgium.
c) General, Organic and Biomedical Chemistry, NMR and Molecular Imaging Laboratory, University of Mons, Place du Parc 20, 7000 Mons, Belgium.

Materials. 12-Hydroxydodecanoic acid (HDA, Aldrich, 97%) and (R,S)-benzyloxycarbonyl-3,3-dimethyl-2-oxetanone (dMMLABn, KymiaNova) were dried by three successice azeotropic distillations by addition of toluene. 1,5,7-Triazabicyclo-[4.4.0]dec-5-ene (TBD, 98%, Aldrich) was dried at 80 °C overnight under vacuum. L-lactide (LLA, GALACTIC, Belgium) was recrystallized three times from dried toluene. DCM solvent was dried using a MBraun Solvent Purification System (model MB-SPS 800) equipped with alumina drying columns. Chemicals were stored and manipulated in a glovebox (H_2O < 3ppm, O_2 < 1ppm)

Characterizations. ^{1}H NMR spectra were recorded using a Bruker AVANCEII-500 apparatus at r.t. in $CDCl_3$ (130mg/0.6ml). NMR DOSY experiments were recorded on an AVANCEII 600 spectrometer equipped with a superconducting magnet of 14.1 T (Bruker, Karlsruhe, Germany) at 25 °C. Bipolar gradient pulses with two spoil gradients were used to measure the diffusion coefficients (BPP-LED pulse sequence). The value of the gradient pulse length δ was 2 ms (for cyclo-PLLA) and 4 ms (for linear PLLA), while the value of the diffusion time Δ was set to 600 ms (for the cyclo-PLLA) and 500 ms (for the linear PLLA). The pulse gradients were incremented in 16 steps from 2% to 98% of the maximum gradient strength (53.5 G/cm) in a linear ramp and the temperature was set at 25 °C. The DOSY spectra were obtained from the TOPSPIN 4.4.1 software and diffusion curves were extracted on 2 different peaks of each studied structures to measure precisely their respective diffusion coefficients. In each case, the mono-exponential diffusion curves were fitted with the well-known equation 1 to extract the diffusion coefficients. The averaged diffusion coefficient was then calculated for the polymer based on these 2 measurements.

$$I = I_o \exp[-\gamma^2 g^2 D \delta^2 (\Delta - (\delta/3) - (\tau/2))] \qquad \text{(equation 1)}$$

where I_0 is the intensity at 0% gradient, γ the gyromagnetic ratio, g the gradient strength, D the diffusion coefficient, δ the gradient pulse length, Δ the diffusion time and τ the interpulse spacing in the BPP-LED pulse sequence.

Size exclusion chromatography (SEC) was performed in THF at 35 °C using a Triple Detection Polymer Laboratories liquid chromatograph equipped with a refractive index (ERMA 7517), a UV detector (254 nm), a capillary viscometry, a light scattering RALS (Viscotek T-60) (Polymer Laboratories GPC-RI/CV/RALS) and an automatic injector (Polymer

Laboratories GPC-RI/UV) and four columns : a PL gel 10 µm guard column and three PL gel Mixed-B 10 µm columns (linear columns for separation of MwPS ranging from 500 to 10^6 daltons).

Scanning Probe Microscopy and analysis. The samples were prepared by drop casting a volume of 10 µL of a 50 µg/mL Chloroform solution onto freshly cleaned silicon wafer pieces (of about 1 cm^2). The deposit is then drying over night under a saturated Chlorofrom atmosphere (i.e. solvent annealing). The acquisition wer done on a Bruker Dimension Icon equiped with a Nanoscope 6 Controller using the PeakForce Tapping mode. RTESPA-300 tips from Bruker Nanoprobe with a resonance frequncy of 300 kHz, a spring constant of 40 N/m, and a tip radius of 10 nm. The image analyses were done with the software Mountains 11 from DigitalSurf (Besançon, France). The automated circle detection process was used to detect the round shaped objects on the images. This analysis is presented here by only showing the particles with the color corresponding to their heigth. Knowing the tip radius and the height of the rings, the actual diameter of the rings can be deduced by considering tip convolution correction.

General polymerization protocol. In a glove box, a dried vial (1) equipped with a magnetic stir bar was charged with 1,5,7-triazabicyclo-[4.4.0]dec-5-ene (TBD, 50.7 mg ; 3.64 x 10^{-4} mol), 12-hydroxydodecanoic acid (HDA, 75 mg ; 3.47 x 10^{-4} mol), and DCM (1.31 g). The mixture was stirred for 10-20 minutes to generate the soluble HDA/TBD salt. In a second vial (2), a solution of L-lactide (LLA, 200 mg ; 1.39 10^{-3} mol) in DCM (3.38 g) was prepared. After a few minutes, 20 µL of the HDA/TBD stock solution was added to the vial (2), corresponding to $[LLA]_0$ = 0.05 M and a $[LLA]_0/[HDA]_0/[TBD]_0$ ratio of 200/1/1.1. After 105 minutes, a 5 µL aliquot was withdrawn outside the glovebox, evaporated under vacuum in a SEC vial, dissolved in THF, and analyzed by SEC to determine conversion and molar mass (Conv. = 85%; relative M_nSEC = 45,000 g/mol; $Đ_M$ = 1.16). In the glovebox, dried (R,S)-benzyloxycarbonyl-3,3-dimethyl-2-oxetanone (dMMLABn, 3 equiv. relative to HDA, 5 µL) was added to the polymerization medium. After 60 minutes, the reaction mixture was precipitated into 7 volumes of heptane, filtered, and dried under vacuum until constant weight. The product was characterized by SEC, ^{1}H NMR, and SPM.

Supplemental Figures

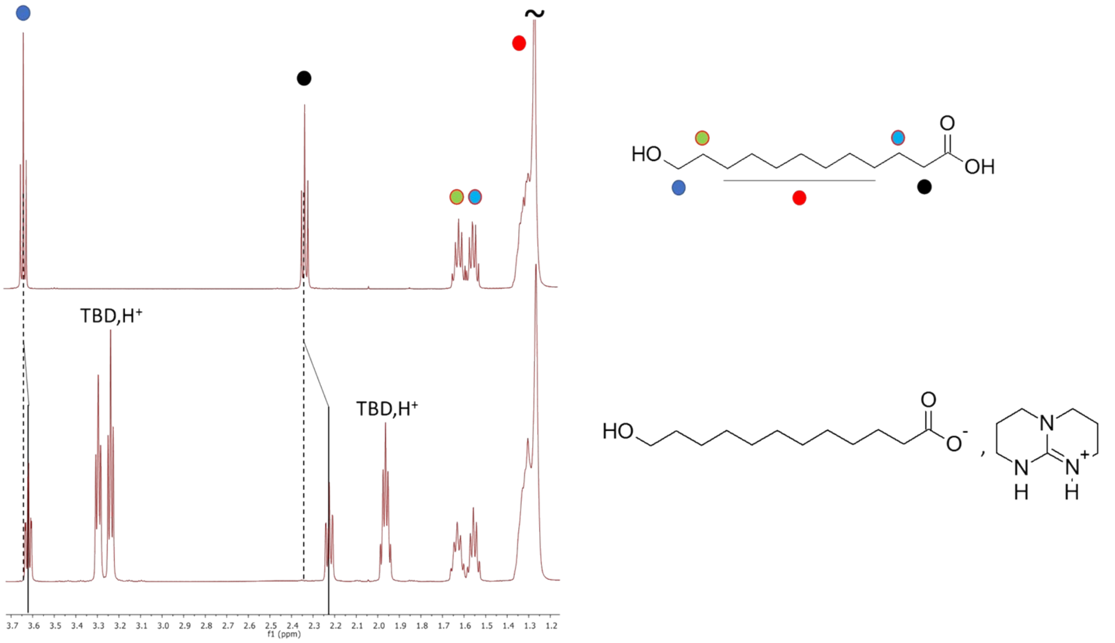

Figure S1. ^{1}H NMR spectra of HDA (top) and an equimolar mixture of HDA and TBD (bottom) recorded in $CDCl_3$ at 21°C (500MHz)

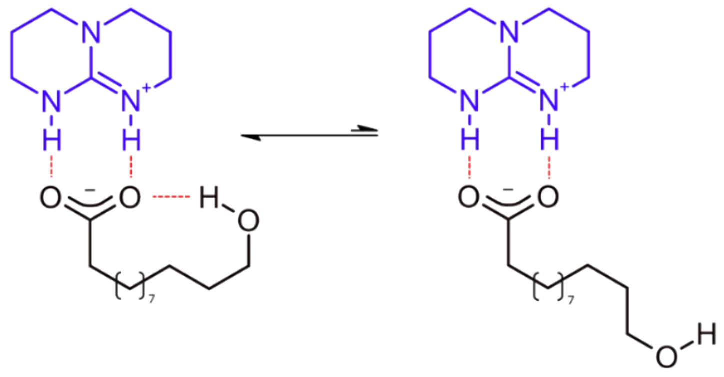

Figure S2. Proposed equilibrium between a preorganized pseudo-cyclic TBD/HDA complex and its open conformation, illustrating the proximity of the hydroxyl group to the ion-pair centre.

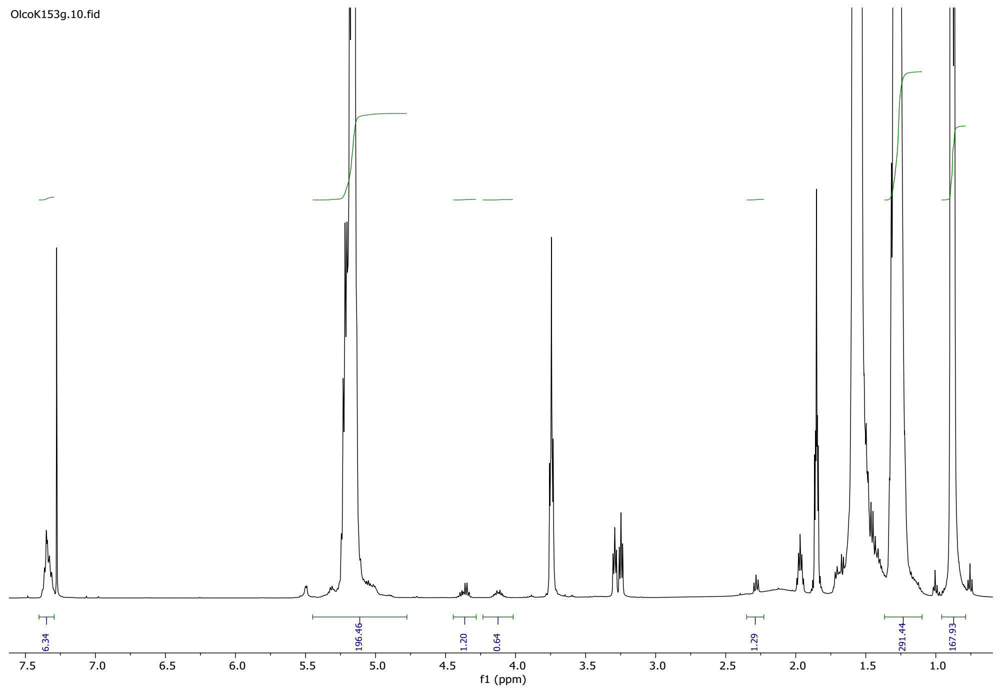

Figure S3. ^{1}H NMR spectrum of *cyclo*-PLLA (M_n = 25,850 g/mol) recorded in $CDCl_3$ at 21°C (500MHz). This spectrum corresponds to Figure 4 and highlights the integration values associated with the assigned signals resonances.

Calculation of β-lactone incorporation and the relative percentage transesterified by the hydroxyl end-group.

From integration values, the following calculations can be made:

1) <u>Number of β-lactone units on the cyclic structure:</u>

- $I_{0.88}$ ≡ 167.96 = 6*H(residual heptane)
- $I_{1.27}$ ≡ 291.44 = 6*n*H(β-lactones) + 10*H(residual heptane)

Thus, 6*n*H(β-lactones) = 11.56

- $I_{4.37}$ ≡ 1.2 = 2*H(HDA) → 6*H(HDA) = 3.6

Therefore, n = $\frac{6.n.H(b-lactone)}{6.H(HDA)} = \frac{11.56}{3.6} = 3.2$

This corresponds to ~ 3 β-lactone units.

2) <u>Percentage of β-lactone units transesterified by the hydroxyl end-group of the PLLA:</u>

We have 6*n*H(β-lactones) = 6*3*H(β-lactones) = 18*H(β-lactones) = 11.56.

If none of the β-lactone units were transesterified, the benzylic aromatic signal at 7.35 ppm, corresponding to 5*n*H(β-lactones) = 15*H(β-lactones) should be equal to 11.56*15/18 = 9.66.

Since $I_{7.35}$ = 6.34, the fraction of transesterified benzyl group is: 1 – 6.34/9.66 = 0.35 → 35%.

Knowing that 3 repeating β-lactone units are present, 3*0.35 = 1.05 ~ 1 benzyl ester dandling group has been transesterified, confirming a 100% efficient ring-closure.

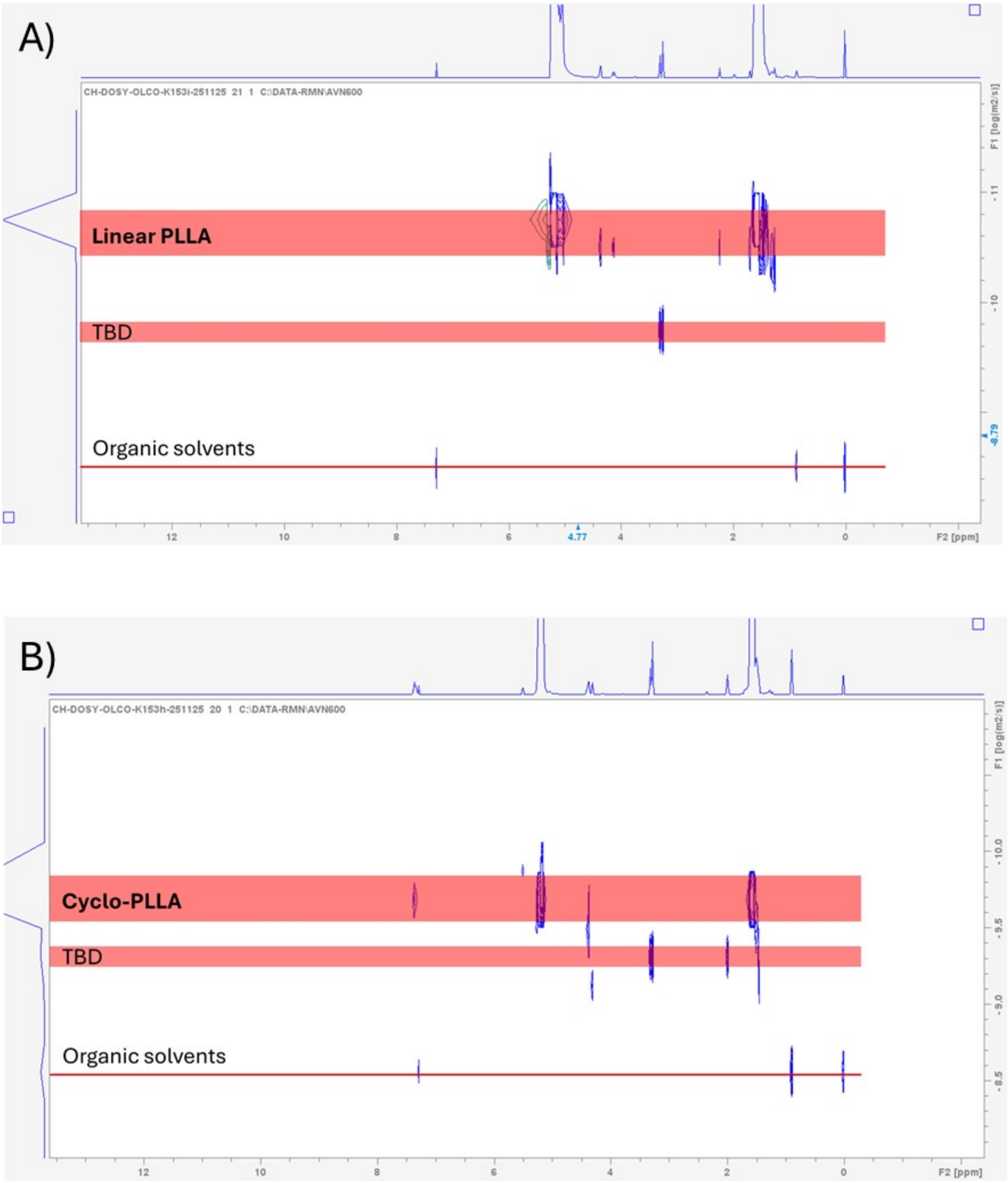

Figure S4. DOSY spectra recorded on linear (A) and cyclo-PLLA (B) solutions in $CDCl_3$ (40 mg/0.6 mL).

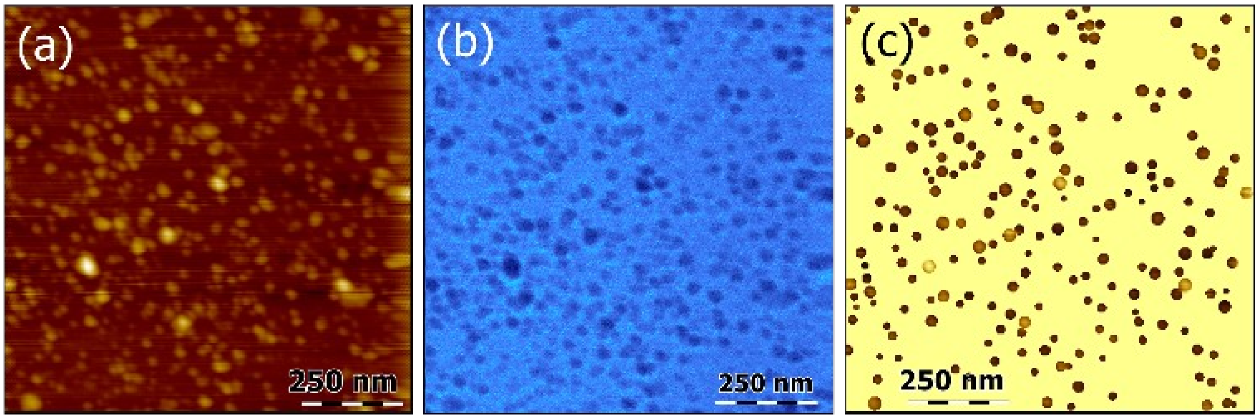

Figure S5. Scanning Probe Microscopy (SPM) acquisitions of a PLLA deposit from chloroform solution (50 μg/mL): (a) topography, (b) adhesion map, and (c) results of automated particle analysis. Scan size: 1.0 μm
